# Combinatorial motif analysis of regulatory gene expression in *Mafb* deficient macrophages

**DOI:** 10.1186/1752-0509-5-S2-S7

**Published:** 2011-12-14

**Authors:** Mariko Morita, Megumi Nakamura, Michito Hamada, Satoru Takahashi

**Affiliations:** 1 Department of Anatomy and Embryology, Institute of Basic Medical Sciences, Graduate School of Comprehensive Human Sciences, University of Tsukuba, 1-1-1, Tennodai, Tsukuba, 305-8575, Ibaraki, Japan

## Abstract

**Background:**

Deficiency of the transcription factor MafB, which is normally expressed in macrophages, can underlie cellular dysfunction associated with a range of autoimmune diseases and arteriosclerosis. MafB has important roles in cell differentiation and regulation of target gene expression; however, the mechanisms of this regulation and the identities of other transcription factors with which MafB interacts remain uncertain. Bioinformatics methods provide a valuable approach for elucidating the nature of these interactions with transcriptional regulatory elements from a large number of DNA sequences. In particular, identification of patterns of co-occurrence of regulatory *cis*-elements (motifs) offers a robust approach.

**Results:**

Here, the directional relationships among several functional motifs were evaluated using the Log-linear Graphical Model (LGM) after extraction and search for evolutionarily conserved motifs. This analysis highlighted GATA-1 motifs and 5’AT-rich half Maf recognition elements (MAREs) in promoter regions of 18 genes that were down-regulated in *Mafb* deficient macrophages. GATA-1 motifs and MafB motifs could regulate expression of these genes in both a negative and positive manner, respectively. The validity of this conclusion was tested with data from a luciferase assay that used a *C1qa* promoter construct carrying both the GATA-1 motifs and MAREs. GATA-1 was found to inhibit the activity of the *C1qa* promoter with the GATA-1 motifs and MafB motifs.

**Conclusions:**

These observations suggest that both the GATA-1 motifs and MafB motifs are important for lineage specific expression of *C1qa*. In addition, these findings show that analysis of combinations of evolutionarily conserved motifs can be successfully used to identify patterns of gene regulation.

## Introduction

In recent years, genomic analyses have identified many short DNA sequences that function as transcriptional regulatory elements and also show evolutionary conservation. These signature sequences are usually referred to as ”motifs”. There is considerable interest in these motifs because variations in gene expression play crucial roles in many biological functions and are also of importance in disease etiology. Much of the information on the roles of these motifs has been obtained using microarray or qRT-PCR analyses to investigate the dynamics of gene expression. These technologies also provide insights into variation in cell fate decision, such as (re)differentiation or (dys)function. The genomic elements that control variations in gene expression, and hence cell fate, are those associated with transcription factors. As a consequence, considerable efforts are being made to detect and characterize these motifs.

Three procedures are widely employed to identify motifs: sequence alignment, motif extraction and motif search. Sequence alignments make it possible to identify biologically meaningful regions [1][2]. In order to expedite investigation of long and complex mammalian genomes, it was necessary to develop computer science methods that permitted analyses to be performed in real-time with high-sensitivity and high-precision. One such approach is the Smith-Waterman algorithm [3], which permits sequence ambiguity, but also provides high accuracy and flexibility, although not with high processing speed. Subsequent development of this algorithm led to the Smith-Waterman-Gotoh (SWG) method, which overcame these problems in efficiency [4]. The SWG method is now the standard algorithm for optimal local alignment, which is a representative method in sequence analysis. The PRRN algorithm, which provides one of the highest-precision approaches, uses a doubly nested randomized iterative (DNR) method for efficient production of multiple alignments [5-7]. Once the multiple alignments have been produced, it is then necessary to undertake motif extraction from conserved common patterns in the set of consensus sequences. Two main methods can be used for this step, namely, the numeration method and the probabilistic method, based on the Weeder [8] and MEME [9] algorithms, respectively. Motif research is also performed to find already-known motifs, for example, using the MAST [10], TFSEARCH [11] and TFBIND [12] algorithms.

Many approaches have been employed to investigate motif interaction, such as regression methods [13]. However, very few studies have focused on regulatory interactions in a large-scale combination of motifs. One such study made use of the Log-linear Graphical Model (LGM) [14] for statistical tests and estimations of the causal relationships among motifs [15]. The LGM is a multivariate analysis and probabilistic model presented as a graph model with probabilistic conditional independency. However, volume of many motifs may be beyond the scaling limits of real-time calculation. It is important to remember, therefore, that current methods require selection of motifs. Additionally, it is important that conclusions derived from computer analyses of interactions among motifs are confirmed by practical methodologies.

With regard to methods for confirmation of interactions, the use of knockout (KO) mice or induced overexpression of transcription factors can provide evidence with a high confidence level. However, these methods generally focus on only one transcription factor at a time. Interactions involving two or more motifs are difficult to identify due to complications arising from motif ambiguity. In our laboratory, we have generated *Mafb* KO mice [16]. MafB is a transcription factor and is known to regulate genes that are expressed in macrophages, a type of leukocyte, in almost all species. MafB is a DNA binding protein with an acidic domain, a basic region and a leucine zipper structure (b-zip structure); it can form a homodimer or a heterodimer with a b-zip structure protein. The protein binds to the Maf recognition element [17,18] and 5’AT-rich half-MARE [19] (MAREs) in regulatory promoter regions. However, despite many investigations, little is known about how MafB achieves regulation of the expression of target genes, or of the cooperation of MafB with other transcription factors. This uncertainty could be surmounted in a timely and cost-effective manner by use of motif detection protocols to sift through large amounts of DNA sequence data.

In this study, we showed bioinformatics methods to identify regulatory motifs and transcription factors which interact with MafB. Through use of the LGM after multiple alignments for finding evolutionarily conserved motifs from a large number of DNA sequences, the relationships of several functional motifs were investigated. In an attempt to understand the motif information, we focused on our analysis of MafB, which is bound to the MARE in the promoter region of genes that are down-regulated in *Mafb* deficient macrophages. The relationships among several motifs were elucidated using data from prior biochemical experiments and from a new study. The observations also suggest that combinations of evolutionarily conserved motifs can be used to predict gene regulation. These techniques for investigating combinations of regulatory motifs in evolutionarily conserved regions should help to accelerate development of applications in medical sciences and lead to elucidation of causes of diseases.

## Methods

### Three approaches for the input sequences

Three approaches were employed for input sequences to discover motifs among multiple sequences [20]. *Multiple genes*, *single species:* this is based on the supposition that regulatory motifs are conserved among co-regulated genes within a species, and that the level of gene expression is constant under the chosen experimental conditions. Different transcription factors might have an indirect influence on the same function. *Single gene*, *multiple species*: the rate of mutation of a regulatory motif is presumed to be slowed by selective pressure. In a single gene group, therefore, universally conserved regions contain regulatory motifs among cross-species. Conserved regions among closely related species may contain less-functional motifs as noise. On the other hand, alignment can be problematic due to changes in function during evolution across species with large evolutionary distances. *Multiple genes*, *multiple species:* alignment of orthologous sequences is used to identify conserved regions; these regions are then analyzed as above in *Multiple genes and single species*. Since potential scores for motif predictions can be improved by alignment of multiple genomes [21], this approach here was adopted to detect regulatory motifs.

### The major steps of the analysis

**Figure 1:**
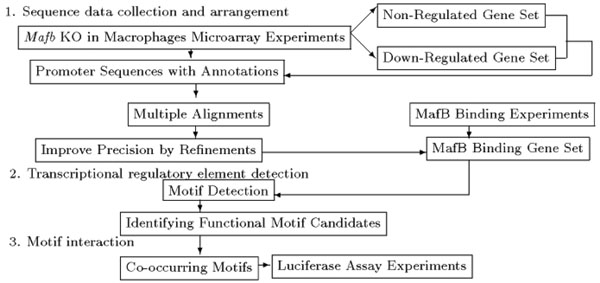
A flow chart of the three methods Three major steps of the analysis are involved in the methodology used.

Three major steps are involved in the methodology used here (Figure 1).

#### Step1-1; Sequence data collection and arrangement

Gene expression data from a previous study were used to identify the promoter sequences of MafB target genes. As *Mafb* KO mice die shortly after birth, embryonic tissues were screened. Microarray (GEO, http://www.ncbi.nlm.nih.gov/geo/query/acc.cgi?acc=GSM511101) and qRT-PCR data were obtained from a set of three independently cultured macrophages from *Mafb* KO mouse fetal liver at embryonic day 14.5 [22]. The DNA microarray analysis was outsourced to JGS Co., Ltd. The National Center for Biotechnology Information HomoloGene database (NCBI, http://www.ncbi.nlm.nih.gov/homologene) was then used to find orthologues of the identified down-regulated and non-regulated genes in 6 mammalian model species. Promoter sequences -2000 bp upstream and +300 bp downstream from each of the transcription start sites were extracted from NCBI and DBTSS [23] core nucleotide databases, using the annotated mRNA Reference Sequence in FASTA format.

#### Step1-2; Identification of consensus sequences by multiple alignments

In order to avoid aligning sequences with an extremely low level of similarity, pairwise alignments were firstly made with the SWG program using data from the mouse and other species with default parameters. Species with a score value of more than 200 and the consensus length of 500bp to 1000bp were selected. Multiple sequence alignments were then carried out for these selected species using SWG, following a progressive method. The PRRN program was then used to refine the alignments to improve the precision of those with lower sequence similarities. Non-conserved regions were masked for possible loss of functions by substitution using the letter ”N” in the nucleotide sequences, the mouse sequences were then collected.

#### Step1-3; Motif extraction for finding MafB binding genes

MafB binding genes in down-regulated genes were first screened using nine short sequences previously confirmed by biochemical experiments [19]. Four categories of consensus motifs, termed here ”MafB motifs”, were generated by the MEME program; nucleotide lengths of N=8, N=9, N=10, and N=11 were used since it is known that an AT-rich sequence is located about three nucleotides upstream of the 6bp MARE. The parameters were set to *allow sites on* + *or - DNA strands; revcomp* and *distribution of motifs; zoops*. Next, each mouse consensus sequence was searched for MafB motifs by the MAST program of the MEME suite. Thus, genes with MafB motifs conserved at least in man and mouse were selected; these genes were named the ”MafB binding gene set”.

#### Step2-1; Motif extraction from the MafB binding gene set

At Step 1-3, MafB motif extraction was performed using nine short sequences previously confirmed by biochemical experiments. Motif extraction was then performed again using MEME to identify other consensus motifs among mouse sequences of the MafB binding gene set with the MafB motifs. Parameters were set to *allow sites on* + *or - DNA strands; revcomp* and *distribution of motifs; zoops*. To aid efficient capture by MEME, nucleotide motif lengths were assigned as N=6,8,10,12,14,16, or 18, because motifs are generally 6~20bp. Longer-length motifs may be palindromes. The ten most significant motifs for each of the 7 length variants were extracted.

#### Step2-2; Search for consensus motifs for transcription factor binding sites

Motif search programs were performed for the 70 motifs extracted at Step2-1 and the 4 MafB motifs at Step1-3 with the TFSEARCH and TFBIND algorithms, which are libraries of preexisting motifs for transcription factors. The options for the TFSEARCH were set as *matrix; vertebrate* and *the threshold value; more than 65* because of using only highly conserved regions. These conditions showed great promise as similar results were obtained with TFBIND. For different tops obtained by each algorithm, the rule that *the two tops were taken when the top of two transcription factors were the same with TFSEARCH and TFBIND*, *and that only the first top was required from each when the top was different with each algorithm* was applied.

#### Step2-3; Identifying functional motif candidates

Among motifs for transcription factors, candidate regulatory motifs, the so-called ”functional motifs” were sought. An Over-Representation Index (ORI) score [24] to identify over-represented motifs in a group was calculated as ,  where *Patt_p_* is the number of a kind of motif present in the down-regulated gene promoters, *Patt_np_* is that in the non-regulated gene promoters, *N_p_* is the number of promoters with a motif in the down-regulated genes and *N_promoter_* is the total number of promoters in the down-regulated genes. A high ORI score indicates that the motif is present evenly among all promoters of down-regulated genes, while a low score indicates that the motif occurs many times in a part of promoters of down-regulated genes. The number of each motif presence in a promoter was then counted with using Weeder-motif locator program, with options as *Minimum match percentage; 90 percent*, *Search the motif in both strands; check*, *Maximum number of substitutions; substitutions: 1 ; N*=*6*,*8*,*: 2 ; N*=*10*,*12*, *: 3 ; N*=*14*,*16*, *: 4 ; N*=*18*. The motif detection algorithm is different to MEME, allowing ambiguous motifs to be obtained in addition to those identified by MEME. Peak ORI scores were labeled ”functional motif candidates”; these sequences contained a MafB motif(Step1-3).

#### Step3-1; Directional relationships of functional motifs

Using the results from the Weeder-motif locator, the patterns of the top motifs by ORI in each promoter were assigned as ”2” when present and ”1” when not present. The patterns were input into the L-GM program [25] to search for directional interactions among the functional motifs. A model was evaluated objectively with deviance and *p-value* for Reduced Model(*RM_t_*) by a backward elimination method from Full Model(*FM*) and it was also presented as an independent graph model with edges and lines. Several combinations of directorial motifs were shown by the final model, and they were checked in all genes.

#### Step3-2; Modeling for the co-occurrence of functional motifs and validation of the hypothesis

A hypothesis and regulatory modeling were derived from the results of the analysis of directional relationships between the MafB motifs and other motifs. The MafB motifs were compared with the results of biochemical experiments to determine whether MafB could actually bind to them to regulate transcription. Validation of the hypothesis was also tested by the results of a luciferase assay using the *C1qa* gene promoter.

## Results

### Collection and multiple alignment of promoters of MafB target genes

The outsourced microarray analysis identified 51 down-regulated genes that showed less than half of the wild type expression level; the value ranges for their mRNA expression were 494 < gProcessedSignal < 169099 (gProcessedSignal; signal intensity of wild type, rProcessedSignal; signal intensity of *Mafb* KO). The microarray did not identify *Emr1*(*F4/80*) as being down-regulated due to an error; however, a previous study showed this gene did show reduced expression [16]. A qRT-PCR analysis showed reproducible results for *Emr1* and some other genes, such as *C1qa*, *C1qb* , *Gas6*, *Adamts1*, and *CD5L*. Therefore, *Emr1* was added to the list of down-regulated genes to give a final total of 52 (Table 1). No change was found in the expression of 211 genes (rProcessedSignal/gProcessedSignal =1.0), with values for mRNA expression close to the minimum or maximum values of the down-regulated group (471 < gProcessedSignal < 229536) because of the large number. The promoters of the down-regulated mouse genes and their orthologues were identified from DBTSS: *Mus musculus*, 52 promoters; *Homo sapiens*, 47; *Pan troglodytes*, 4; *Rattus norvegicus*, 34. From NCBI, : *Pan troglodytes*, 1 promoter; *Bovine*, 31; *Canis familiaris*, 6; *Rattus norvegicus*, 10 were obtained. The promoters of the 211 non-regulated genes and of the orthologues were obtained from NCBI. After multiple alignments of the orthologues, they were refined and improved in the precision of alignments. Non-conserved regions were masked, then mouse sequences were collected. In order to select MafB binding genes among the 52 down-regulated genes, 4 categories of ambiguous motifs named ”MafB motif” were extracted from 9 MafB binding sequences confirmed by biochemical experiments(Table 2). In the set of 52 down-regulated genes, 39 were found to have *E-values* for the MafB motif of less than 21 in the MAST program. Of these, 18 genes remained after removing those with no conservation between man and mouse; these 18 genes formed the ”MafB binding gene set”(Table 3).

**Table 1:** Fifty-two down-regulated genes in macrophages from *MafB* deficient mice

*Lbp*, *Slc43a3*, *Rtp4*, *Gdf15*, *Isg15*, *Lgals3bp*, *Mafb*, *Psd3*, *Chst7*, *Col18a1*, *C1qa*, *Bambi*, *Slc9a3r1*, *Glt25d1*, *Ifit3*, *Tmem66*, *Ifi44*, *Usp18*, *Clu*, *Gad1*, *Ndrg4*, *Cnrip1*, *Folr2*, *C1qb*, *Trib3*, *Daf2*, *Htr2b*, *Irf7*, *Bst2*, *Leprotl1*, *Adamts1*, *Cxcl10*, *Igfbp1*, *Ifit1*, *Rbp4*, *Rab15*, *Cd55*, *Gas6*, *Ifit2*, *Defb29*, *Setd2*, *Iigp1*, *Gatad2a*, *Ccl12*, *Krt18*, *1810011O10Rik*, *Fgb*, *Phgdh*, *Hpgd*, *Ambp*, *Cd5l*(*Api6*), *Emr1*(*F4/80*)

**Table 2:** Four patterns of predicted MafB motifs

MafB binding sequence	MafB motif		
tgtctatgctcag	**Width**	**Motif**	**Ambiguous motif**
cttttgtgctgtt	N=8	CTGCTGAC	[CTG]TGCT[GC]AC
ccaaactgctgac	N=9	TCTGCTGAC	[TAG][CTG]TGCT[GC]AC
cgtaactgctgac	N=10	ATCTGCTGAC	[AT][TAG][CTG]TGCT[GC]AC
caaatttgcagac	N=11	TATCTGCTGAC	[TA][AT][TAG][CTG]TGCT[GC]AC
taaagttgctgaa	(N=Nucleotide)		
catttctgctgac			
aggatgtgatgac			
tgttgttgctcac			
(Underline; MARE)			

MafB motifs were extracted from 9 short MafB binding sequences containing 5’ AT-rich half-MARE(MARE) and validated by biochemical experimentations.

**Table 3:** Eighteen predicted MafB binding genes

*Adamts1*, *Ambp*, *Bambi*, *C1qa*, *C1qb*, *Cd5l*(*Api6*), *Chst7*, *Clu*, *Cxcl10*, *Emr1*(*F4/80*), *Gad1*, *Gas6*, *Igfbp1*, *Krt18*, *Lbp*, *Mafb*, *Slc43a3*, *Slc9a3r1* [ NCBI Accession Number (respectively): NM_009621, NM_007443, NM_026505, NM_007572, NM_009777, NM_009690, NM_021715, NM_013492, NM_021274, NM_010130, NM_008077, NM_019521, NM_008341, NM_010664, NM_008489, NM_010658, NM_021398, NM_012030 ]

### Identification of 10 functional motif candidates

In total, 70 consensus motif seeds were generated from the set of 18 MafB binding genes by motif extraction, and were given individual ID numbers. The 70 motifs and the 4 MafB motifs were input into TFSEARCH and TFBIND. The best binding to each motif was found in 2 types of transcription factors at most. Next, ORI scores were calculated for all 74 seeds, and the top 10 motifs with the highest scores were selected as functional motif candidates; these candidates, their transcription factors and IDs in rank order are shown (Table 4). All 10 candidates were known motifs and had score values of over 61. The scores were high because it could be meaningful in general if a motif had a score value of more than 1 [24]. Of the top 10 motifs, ”10 AP4/AML ATCTGCTGAC” from Step1 and ”10-10 Nkx-2.5 GTCTGAGTG” from the MafB binding gene set contained MARE, therefore, they were identified as MafB motifs with a high probability of MafB binding.

**Table 4:** Ten functional motif candidates

Group:Motif ID	TF	Motif (ORI ≥ 61, Underline; MARE)	
E:12-7	MYOD, GATA-1	CAGCCAGCAGCA	
G:14-4	CdxA	AGCTTTGAAGACAG	
F:16-2	GATA-2, AP2	CGGTCTCTGGGCTCAG	(Partially same strand of 14-2)
A:10	AP4/AML(10ATCT..)	ATCTGCTGAC	Only group ”A” is extracted at Step1.
F:14-2	AP2/ZID	CGGGCTCTGGGCTC	
F:,B:12-2	AP2, AML-1a	AGCCCAGAGCCC	(Complementary strand of 14-2, 16-2)
D:10-10	Nkx-2.5	GTGCTGAGTG	
E:10-3	GATA-1	CCTGCTCCTG	(Complementary strand of 12-7)
B:16-4	AML-1a	ACAGAGGCCCAGAGGG	(Partially same strand of 12-2)
C:10-9	MZF1	GCTGGGGCAG	

The top 10 functional motif candidates were optimized by ORI and are shown with their transcription factors and the IDs in the order of their score ranking. The IDs show both the number of nucleotides and the ranking order of extracted motifs, for example, the ID of the seventh extracted motif using N = 12 is ”12-7”. The motifs were grouped by sequence similarity with the same transcription factor. Two motifs, groups A and D, were identified as the MafB motif.

### Co-occurrence of functional motifs

#### Occurrence patterns of functional motif candidates in all 229 genes

The presence of the 10 functional motif candidates were examined in 229 gene promoters (the 18 down-regulated and the 211 non-regulated genes) to investigate the co-occurrence of functional motifs. A large gene dataset is valuable for this type of investigation as it enables to compare the patterns of occurrence of motifs in both down-regulated and non-regulated genes. The occurrence of a motif was labeled as ”2”, its absence as ”1”. Most motifs fell into the former category in the down-regulated genes, but into the latter in the non-regulated genes. Several motifs showed a similar pattern in all 229 genes when they were (reverse) complementary with the same transcription factor, as (IDs;10-3, 12-7), (IDs;12-2, 16-4) and (IDs;12-2, 14-2, 16-2). The motif pattern data were combined by similarity, except MafB motif [10 AP4/AML ATCTGCTGAC] from Step1-3.

#### LGM for 10 combinations of functional motif candidates

**Figure 2:**
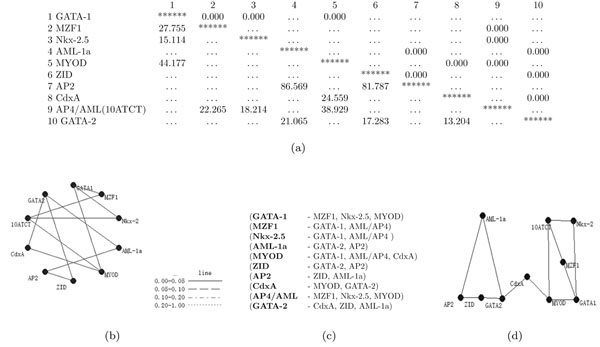
Final model and 10 motif combinations (a) ***p-value* and deviance** The lower left diagonal of the table (left triangle) shows deviances, and the right upper (right triangle) shows *p-values* in the Table of the final model. (b) **An independent graph** When the final model was selected (*p-value* < 0.001), the independent graph indicates several directional interactions of motif candidates. (c) **Motif combinations** Ten motif combinations were selected. (d) **Motifs in the independent graph **Position of motifs in the independent graph were arranged to provide an intuitive display. The graph shows that MYOD, GATA-1 and MZF1 interact directly with MafB motifs, as AP4/AML(10ATCT) and Nkx-2.5 motifs.

LGM was used to evaluate the co-occurrence of functional motif candidates. Here, ”10ATCT” instead of AP4/AML (motif ID 10) was written owing to the long length of the motif name. The deviance and *p-value* were also calculated with the defined formula. The inclusion of 10 functional motif candidates increased drastically the number of degrees of freedom, making it difficult to assess a final *RM* by tests based on model deviance. A final model was therefore selected when the *p-value* became lower than 0.001(Figure 2-a). The final model had a deviance of 122.806 at 1001 degrees of freedom and a *p-value* of 1.000 against the *FM*. Linked motifs are indicated by an independent graph (Figure 2-b). Ten combinations of co-occurring functional motif candidates were obtained (Figure 2-c). The positions of the motifs are arranged in the independent graph to enable intuitive understanding of the final model (Figure 2-d). AP2, ZID and GATA-2 motifs appeared to have almost the same sequences or reverse complementary chains, MyoD and GATA-1 motifs were also similar. Motifs regarded as belonging to the same group were positioned transversely. The 10ATCT(AP4/AML) and Nkx-2.5 motifs are MafB motifs. Two groupings were present and were linked by CdxA.

#### Co-occurrence of MafB motif and GATA-1 motif

Genes with a combination of functional motif candidates in their promoters are indicated by a star in (Table 5). Several of the combinations with the GATA-1 motif and MafB motif (underline) showed many stars. The GATA-1 motif had high scores in the ORI calculation; thus this motif, along with the MafB motif, seemed to be important for the regulation of expression of the MafB binding gene set. This suggests that the GATA-1 motif and the MafB motif occur as a pair, and they therefore form a ”Functional motif” as their directional relationship. The motif locations of all the combinations in each down-regulated gene were examined. Since a translation start site that begins with the sequence ”ATG” might be mistaken as a YY1 transcription factor sequence, it was confirmed not to be YY1 by TFSEARCH. The combinations showed a trend that the MafB motif, as AP4/AML or Nkx-2.5 motifs, was closely located or sometimes overlapped to the GATA-1 motif and many of these motifs were located near each transcription start site.

**Table 5:** Combinations of functional motif candidates in each gene

(P < 0.001) Linked motif	GATA-1 MZF1, Nkx-2.5, MYOD	MZF1 GATA-1, AP4/AML	Nkx-2.5 GATA-1, AP4/AML	AML-1a GATA-2, AP2	MYOD GATA-1, AP4/AML, CdxA	ZID GATA-2, AP2	AP2 ZID, AML-1a	CdxA MYOD, GATA-2	AP4/AML MZF1, Nkx-2.5, MYOD	GATA-2 CdxA, ZID, AML-1a
Gene										
*C1qa*		⋆	⋆							
*Chst7*	⋆	⋆	⋆							
*C1qb*	⋆	⋆	⋆	⋆		⋆	⋆			
*Emr1*(*F4/80*)			⋆				⋆			
*Cd5l*(*Api6*)			⋆							
*Ambp*	⋆	⋆	⋆							
*Bambi*		⋆								
*Cxcl10*					⋆		⋆			
*Igfbp1*	⋆	⋆	⋆		⋆				⋆	
*Gad1*							⋆			
*Clu*		⋆			⋆					
*Slc43a3*							⋆			
*Gas6*		⋆								
*Lbp*		⋆								
*Adamts1*				⋆		⋆	⋆			
*Slc9a3r1*	⋆	⋆	⋆	⋆	⋆	⋆	⋆	⋆	⋆	⋆
*Mafb*	⋆	⋆	⋆	⋆	⋆	⋆	⋆	⋆	⋆	⋆
*Krt18*	⋆	⋆	⋆	⋆		⋆	⋆		⋆	

Combinations in which motifs co-occurred are marked by a star ”⋆”.

The underline was MafB motif.

#### Hypothesis and Modeling

**Figure 3:**
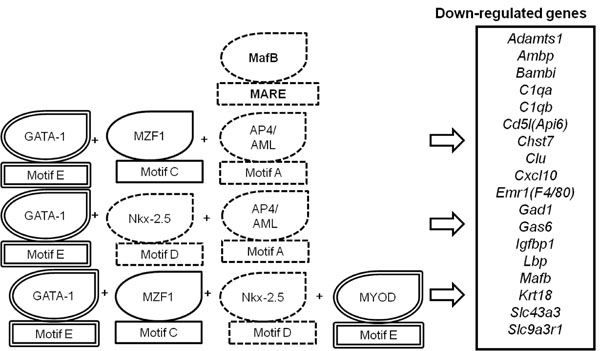
Modeling of the hypothesis A hypothesis on motif combinations was derived from the results. The dotted surrounding line indicates a MafB motif, and the double surrounding line indicates a GATA-1 (or MYOD) motif. Combinations of co-occurring functional motifs [A·C·E], [A·D·E] and [C·D·E](underline; MARE), shown by the stars in Table 5, lead to the negative or positive regulation of MafB target genes expression (Table 4; underline, MARE).

The analysis provided insight into the cooccurrence of the functional motifs required for the regulation of MafB target gene expression. A hypothesis on motif combinations was constructed and the modeling of this hypothesis is shown in (Figure 3). The hypothesis postulates that **”GATA-1 motifs and MafB motifs negatively and positively regulate expression of 18 genes that are down-regulated in *Mafb* deficient macrophages, respectively”.**

### Results of biochemical experiments and hypothesis validation

#### Evaluation of the MafB motif using the results of biochemical experiments

The results from biochemical experiments were used to confirm the binding of MafB to the MafB motif and its regulation of transcription. Two of the mouse genes present in the set of 18 down-regulated genes were subjected to a luciferase assay. Mutations of MARE in *C1qa* and *Cd5l* (*Api6*, *AIM*) promoters considerably impaired promoter activity in RAW264.7 macrophage cells. Cotransfection of the luciferase reporters driven by the gene promoters along with MafB expression vectors dramatically decreased luciferase gene expression.

**Figure 4:**
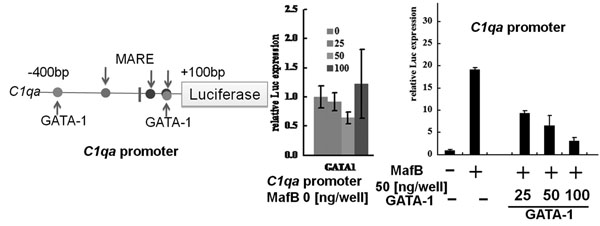
Analysis of *C1qa* by a luciferase assay Plasmids were constructed that carried the pGL4 *C1qa* promoter-luciferase and MafB and GATA-1 motifs. RAW264.7 cells were transfected to express each construct and the effects on luciferase reporter activity were studied. The Single GATA-1 in the *C1qa* promoter had little influence. Inducing MafB increased *C1qa* promoter driven luciferase activity. This activity was inhibited by GATA-1.

The constructs were generated using two oligonucleotides with mutations at -82 bp upstream of the transcription start site in *C1qa* (data not shown), and -54bp upstream of the start site in *Cd5l* (*Api6*, *AIM*)(data not shown). These results indicate that the MARE immediately upstream of the transcription start site is required to regulate *C1qa* and *Cd5l* (*Api6*, *AIM*) promoter activity. Additionally, this confirms that MafB binds to the MafB motif with MARE to regulate transcription, as the MAREs were located in the ID 10-10 and ID 10 motifs. Another luciferase assay was performed with the same experimental methods using *C1qa* promoter to determine whether the GATA-1 and MafB motifs were positioned within 400bp upstream and 100bp downstream of the transcription start site (Figure 4). Overexpression of GATA-1 had little effect on the *C1qa* promoter; in contrast, MafB vector-induced luciferase activity was significantly increased. The GATA-1 vector reduced luciferase activity in a concentration-dependent manner in the presence of MafB. These results demonstrated that the GATA-1 motif of the *C1qa* promoter could inhibit the activity of the *C1qa* promoter, which has the GATA-1 and MafB motifs.

## Discussion

In this study, information from an LGM analysis on the directional relationships of several functional motifs is presented. The results suggest that both GATA-1 motifs and MafB motifs in the promoter region are important for lineage specific expression of genes that are down-regulated in *Mafb* deficient macrophages. It was also possible to validate the conclusions from the LGM analysis using the results of biochemical experiments. Overall, the findings indicate that it is possible to predict combinations of evolutionarily conserved motifs for gene regulation from large-scale DNA sequences. These directional motifs were obtained using objective application of a final model at a *p-value* of < 0.001 (Figure 2-a) and an independent graph by LGM (Figure 2-b). The promoter regions of 15 genes down-regulated in *Mafb* deficient macrophages contained GATA-1 motifs and 5'AT-rich half Maf recognition elements (MAREs) (Table 5). This suggests that both the GATA-1 motif and the MafB motif have a role in the negative and positive regulation of expression of these genes, respectively. The GATA-1 motif had high score in an ORI, possibly indicating overrepresentation of the motif in the down-regulated genes (Table 4). To investigate this possibility a luciferase assay was performed using a *C1qa* promoter that had both the GATA-1 motif and MARE. GATA-1 inhibited the activity of this promoter (Figure 4).

The 4 nucleotides in GATA-1 motif are often the same as the 6 nucleotides of MAREs. This suggests that MafB binding may be prevented by GATA-1, or that both of motifs with these proteins may bind each other for transcriptional or translational repression. GATA-1 and MafB may influence the level of mRNA expression in MafB target genes and thereby cause the generation of abnormal protein levels. As a consequence, defective MafB regulation may underlie abnormal functions in macrophages. GATA-1 has been shown to suppress monocyte differentiation in bone marrow, and to inhibit macrophage differentiation and apoptosis [26,27]. These motifs therefore regulate gene expression patterns to support the direction of macrophage differentiation. In hematopoietic stem cells, GATA-1 activates HDAC(histone deacetylase) and down-regulates the GM-CSF (Granulocyte Macrophage Colony-Stimulating Factor) gene to promote differentiation of erythrocytes [28]. However, since GATA-2 and MZF-1 down-regulate HDAC, they are thought to be related to hematopoietic differentiation. In erythroid progenitor cells, GATA-2 acts with MZF1 to suppress macrophage differentiation, while the loss of GATA-1 results in up-regulation of GM-CSFR and differentiation into macrophages. GATA-1 and MZF1 are therefore considered to be related to macrophage differentiation, and the cooccurrence of MZF1 and GATA-1 motifs are likely to influence macrophage differentiation.

The GATA-1 is generally required for the differentiation and proliferation of erythrocytes, and the MafB is known to be essential for maintaining macrophage function. The co-occurrence of GATA-1 and MafB motifs initially seems to be contradictory with respect to macrophages. However, it has been reported that in macrophages MafB is a repressor of Ets-1, an inhibitor of erythrocyte differentiation in the chicken [29]. Another study showed that ectopic expression of GATA-1 in myeloid cells increases erythrocyte differentiation [26]. When Ets-1 is expressed in both erythroid and myeloid cells, MafB expression is limited to the latter [26]. Thus, MafB is an essential factor for differentiation or functional maintenance of myeloid cells. However, the decrease of MafB expression is mediated by Ets-1, and the expression of GATA-1 reactivates erythrocyte differentiation. This effect was presumed to be a result of the loss of MafB that stimulated GATA-1 to change the direction of differentiation. These observations also support the hypothesis that MafB and GATA-1 motifs co-occur in macrophages. MafB deficiency may enable cells to re-differentiate erythrocytes from macrophages. It will be necessary to determine whether the MafB and GATA-1 motifs in macrophages are involved in erythrocyte differentiation. Deficiency of GATA-1 allows erythroid cells to re-differentiate into macrophages [30-33]. If the identified GATA-1 motif of macrophages is also present in erythroid cells, then it may be capable of inducing the re-differentiation of macrophages to erythrocytes. Furthermore, ectopic expression of C/EBP*α*, GATA-1, or GATA-2 strongly enhances the macrophage differentiation potential of Pax5 KO pro-B cells under lymphoid culture conditions, whereas GATA-1 expression induces erythroblast development [34,35](MACPAK: Simulatable Macrophage Pathway Knowledgebase database, http://macpak.csml.org/click/index.php?q=d:18472258). The transdifferentiation of pro-B cells to macrophages or erythrocytes may be determined by their MafB and GATA motifs. Thus, through investigation of co-occurring motifs it may be possible to identify the switches for cell differentiation.

## Conclusions

Identification of combinations of transcriptional regulatory elements from large amounts of DNA sequences can benefit greatly from proposed bioinformatics methods as the multiple alignments and the LGM. In particular, the pattern of co-occurrence of motifs is proposed to provide a strong means for directing potential interaction of transcription factors not known from biochemical studies. The hypothesis postulates that GATA-1 motifs and MafB motifs negatively and positively regulate expression of 18 genes that are down-regulated in *Mafb* deficient macrophages, respectively. This result was verified experimentally. By combining the bioinformatics analysis and the experimental approach, it was suggested that both the GATA-1 motifs and the MafB motifs are important for expression of *C1qa*. Through the use of the bioinformatics methods, the regulatory motifs related to MafB have been identified and their interactions were suggested. The findings here also suggest that combinations of evolutionarily conserved motifs are capable of predicting gene regulation. It is to be expected that future studies will elaborate on and develop these findings. For example, further research on the control of gene expression may discover rules for the co-occurrence of particular motifs in the genomes. Eventually, these new techniques for identifying regulatory elements should help to accelerate development of applications in medical sciences and lead to elucidation of the causes of different diseases.

## List of abbreviations used

AML1: acute myeloid leukemia 1 gene; AP4: transcription factor ap4; cDNA: complementary dna; *C1qa*: complement component 1, q subcomponent, a chain; DBTSS: database of transcriptional start sites; DNR: doubly-nested randomized iterative; DNA: deoxyribonucleic acid; FM: full model; GM-CSFR: granulocyte/macrophage-colony stimulating factor receptor; GATA: gata-binding protein; HDAC: histone deacetylase; KO: knockout; LGM: log-linear graphical model; Maf: musculoaponeurotic fibrosarcoma; MARE: maf recognition element; MEME: motif-based sequence analysis tools; MZF1: myeloid zinc finger gene 1; N: nucleotides; NCBI: national center for biotechnology information; ORI: over-representation index; RM: reduced model; ZID: zinc fingerprotein with interaction domain; PRRN: multiple sequence alignment program; SWG: Smith-Waterman-Gotoh algorithm.

## Competing interests

The authors declare that they have no competing interests.

## Authors contributions

MM developed the bioinformatics methods, implemented the algorithms, carried out the data analysis, and drafted and wrote the manuscript. MH had made substantial contributions to acquisition of microarray data, and was involved in critical study of important intellectual content. MH designed, MN and MM carried out the experiments. ST conducted the supervision of the study and manuscript preparation, and gave final approval of the version to be published. All authors read and approved the final manuscript.
